# Investigation of salt-bridge interactions underlying salt activation of halophilic methionine sulfoxide reductase A

**DOI:** 10.3389/fmicb.2026.1907662

**Published:** 2026-08-24

**Authors:** Shuhong Chen, Lianghui Liu, Hailang Zhou, Xiaoxue Kuang, Ye Li, Rong Liu, Jiawei Yang, Xiaoling Cheng

**Affiliations:** 1 Department of Biochemistry, School of Preclinical Medicine, Zunyi Medical University, Zunyi, Guizhou, China; 2 Department of Clinical Laboratory, Guizhou Aerospace Hospital, Zunyi, Guizhou, China; 3 Department of Cell Biology, School of Preclinical Medicine, Zunyi Medical University, Zunyi, Guizhou, China

**Keywords:** halophilic enzyme, methionine sulfoxide reductase A, molecular dynamics, salt activation, salt bridge

## Abstract

**Introduction:**

Halophilic microorganisms inhabit hypersaline environments where sustained ionic stress challenges protein structure and function, making salt-responsive enzymes important for cellular fitness. Here, we investigated the salt-bridge interactions that support salt-dependent activation of a halophilic methionine sulfoxide reductase A from *Halobacterium hubeiense* (*Hh*MsrA), a key enzyme involved in repairing oxidized methionine residues.

**Methods:**

Molecular dynamics (MD) simulations, mutagenesis analyses, enzyme activity assays, and circular dichroism (CD) measurements were used to identify key salt-bridge-forming residues and evaluate their contributions to the salt-dependent structural and functional behavior of *Hh*MsrA.

**Results:**

MD simulations and mutagenesis analyses identified Arg106 and Lys76 as prominent salt-bridge nodes, and charge-conservative substitutions at these positions largely preserved the high-salt activity profile, whereas non-conservative substitutions (e.g., acidic, hydrophobic, aromatic, or proline replacements) generally reduced activity. Moreover, combined proline substitutions produced a pronounced cumulative effect, leaving the enzyme with only minimal activity even under high-salt conditions. MD analyses further associated these site-specific salt-bridge interactions with the maintenance of the salt-adapted structural state required for *Hh*MsrA activity. CD measurements further showed a strong KCl-dependent helical response in *Hh*MsrA that was attenuated to varying degrees across mutants targeting salt-bridge-forming residues.

**Discussion:**

These findings demonstrate that salt-dependent activity of HhMsrA is supported by a small set of salt-bridge nodes that contribute to maintaining a high-salt structural state compatible with *Hh*MsrA catalytic function.

## 1 Introduction

Halophilic microorganisms inhabit saline and hypersaline environments in which sustained ionic and osmotic stresses challenge macromolecular stability (Oren, 2024). To maintain cellular functions, halophiles have evolved several physiological strategies, including intracellular KCl accumulation through the salt-in strategy and the synthesis or uptake of compatible solutes such as betaine and trehalose (Ding et al., 2022; Ionescu et al., 2024). Recent evidence further indicates that some halophilic bacteria can adopt hybrid osmoregulatory modes, underscoring the ecological and physiological diversity of halophilic adaptation (Ionescu et al., 2024; Xing et al., 2024). At the protein level, halophilic adaptation is frequently associated with proteome acidification and highly charged surfaces that promote strong hydration and reduce aggregation under concentrated salts, a feature repeatedly highlighted in foundational work and reinforced by recent analyses of electrolyte–protein interactions and extremophile adaptation trade-offs (Oren, 2013; Herrero-Alfonso et al., 2024; Geraili Daronkola et al., 2025). Beyond global surface charge, specific electrostatic contacts can shape conformational landscapes under saline conditions, and salt bridges and ion-pair networks have been implicated as tunable stabilizing elements in enzymes (Nayek et al., 2014; Cui et al., 2021). Consistent with this view, high-salt conditions can favor the conformational stability of haloarchaeal enzymes, thereby maintaining structural states compatible with catalytic function. Ion-specific effects may further influence these conformational equilibria through interactions with charged surface residues (Ortega et al., 2011; Schindl et al., 2024).

Hypersaline environments can also impose oxidative challenges on halophilic microorganisms, and haloarchaea in particular have evolved dedicated protective mechanisms to mitigate oxidative stress (Matarredona et al., 2024). Oxidative stress promotes protein oxidation, and methionine residues are among the most susceptible targets, forming methionine sulfoxide and thereby altering protein structure and function (Ezraty et al., 2017; Vincent and Ezraty, 2023). Methionine sulfoxide reductases (Msrs) constitute a conserved protein-repair system that reverses this modification by reducing methionine sulfoxide back to methionine, and accumulating evidence highlights their roles in protein quality control under stress conditions (Aussel and Ezraty, 2021). In particular, MsrA is stereospecific for the *S*-diastereomer of methionine sulfoxide, whereas MsrB reduces the *R*-diastereomer, providing a complementary enzymatic route to restore methionine in oxidized proteins (Lourenço dos Santos et al., 2018; Kim et al., 2021). Beyond their physiological roles, the high stereoselectivity of Msrs has also been leveraged for the green synthesis of enantiopure sulfoxides, which are valuable motifs in pharmaceuticals and fine chemicals (Peng et al., 2022; Zhao et al., 2023; Jiang et al., 2024; Zhang et al., 2024; Song et al., 2025). For halophilic microorganisms, maintaining MsrA activity under under high-salinity conditions is therefore important for sustaining methionine redox homeostasis and cellular proteome integrity (Aussel and Ezraty, 2021; Matarredona et al., 2024).

We previously characterized a salt-activated methionine sulfoxide reductase A from the halophilic archaeon *Halobacterium hubeiense* (*Hh*MsrA) and identified Arg168 as an important contributor to the maintenance of *Hh*MsrA activity under high-salt conditions through its involvement in salt-bridge interactions (Zhou et al., 2024). Although this finding revealed a site-specific electrostatic contribution to the salt-dependent structural behavior of *Hh*MsrA, whether additional salt-bridge nodes cooperatively support its structural integrity and activity of *Hh*MsrA under high-salt conditions remained unclear. In this study, we expanded the salt-bridge analysis to identify additional candidate residues, evaluated their roles through targeted and combined mutagenesis, and integrated circular dichroism spectroscopy with molecular dynamics simulations to link salt-dependent activity to conformational changes and site-specific electrostatic contacts. Collectively, our results refine the structural model underlying the salt-dependent activity of *Hh*MsrA and provide a framework for understanding how enzymes from halophilic microorganisms couple salinity to catalytic competence.

## 2 Materials and methods

### 2.1 Strains, plasmids, and reagents

The gene encoding methionine sulfoxide reductase A from the halophilic archaeon *Halobacterium hubeiense* strain JI20-1 (*Hh*MsrA) was obtained from our previous work (Zhou et al., 2024). The coding sequence was cloned into the expression vector pET-28a to generate an N-terminal His-tagged construct. *Escherichia coli* strain BL21(DE3) was used for recombinant expression. L-methionine sulfoxide (L-Met-O) and other chemicals were obtained from commercial sources. All reagents were of analytical grade unless otherwise specified.

### 2.2 Site-directed mutagenesis and mutant construction

Site-directed mutagenesis of the *Hh*MsrA gene was performed by PCR using a commercial kit (Sangon Biotech, China), and all primers used in this study are listed in Supplementary Table S1. PCR was carried out with an initial pre-denaturation at 95 °C for 3 min, followed by 30 cycles of denaturation at 95 °C for 30 s, annealing at 65 °C for 30 s, and extension at 68 °C for 3 min. The amplified PCR products were digested with *Dpn* I at 37 °C for 1 h to remove the methylated parental template DNA, and the digested products were then transformed into *Escherichia coli* BL21(DE3) competent cells. Positive clones were verified by DNA sequencing to confirm the intended mutations. Single-site variants were generated at Arg106 (R106K, R106E, R106A, R106P, and R106F) and Lys76 (K76R, K76E, K76A, K76P, and K76F). The combined mutants K76P–R106P and K76P–R106P–R168P were constructed by gene synthesis (Sangon Biotech, China) and sequence-verified prior to expression.

### 2.3 Recombinant expression and purification of *Hh*MsrA and variants

Overnight cultures harboring the expression plasmid were diluted 1:100 into LB medium and grown at 37 °C until the OD_600_ reached 0.6. Protein expression was induced with 1 mM isopropyl *β*-D-1-thiogalactopyranoside (IPTG), and the cultures were incubated at 20 °C for 18 h. Cells were harvested by centrifugation at 4,000 rpm for 10 min and lysed by sonication on ice at 90 W using 1.5-s pulses separated by 2.5-s intervals for a total elapsed time of 5 min. The lysates were clarified by centrifugation at 12,000 rpm for 15 min at 4 °C to obtain crude enzyme extracts. Recombinant *Hh*MsrA and variants were purified by Ni–NTA affinity chromatography through the N-terminal His_6_-tag. Protein concentrations were determined using a bicinchoninic acid (BCA) assay and converted to micromolar (μM) according to the molecular weight of each protein. Protein purity was assessed by sodium dodecyl sulfate–polyacrylamide gel electrophoresis (SDS–PAGE) using a 12% resolving gel. Protein samples were heated at 100 °C for 5 min before loading. Electrophoresis was initially performed at 80 V until the samples entered the resolving gel and was then continued at 120 V for approximately 60 min, until the bromophenol blue dye front reached approximately 0.5 cm from the bottom of the gel. The gel was stained with Coomassie Brilliant Blue for 15 min and subsequently destained. The SDS–PAGE analysis of these proteins is provided in Supplementary Figure S1.

### 2.4 Enzyme activity assay and salt-dependent activity profiles

The specific activities of *Hh*MsrA and its mutants were determined using a dithiothreitol (DTT)–5,5′-dithiobis (2-nitrobenzoic acid) (DTNB) colorimetric coupling assay according to a previously reported protocol (Wu et al., 2013). The specific activity was calculated based on DTT consumption. Briefly, reactions were carried out in a total volume of 500 μL containing 3 μM purified protein, 3 mM methionine sulfoxide substrate (L-Met-O), 1 mM DTT, and KCl at concentrations ranging from 0 to 3.5 M. The reaction mixture was incubated for 45 min under the optimal conditions (pH 10.0, 40 °C). Subsequently, 50 μL of the reaction mixture was transferred to a 96-well microplate, followed by the addition of 150 μL DTNB solution to a final concentration of 2 mM. After incubation at 37 °C for 15 min, absorbance at 405 nm (A_405_) was measured using a microplate reader. A blank control without enzyme was included in parallel. Specific activity was calculated according to the decrease in A_405_, and salt-dependent activity profiles were generated by plotting specific activity against KCl concentration. One unit (U) of enzyme activity was defined as the amount of enzyme required to consume 1 μmol DTT per minute. Specific activity was calculated by normalizing enzyme activity to the amount of enzyme protein and was expressed as mU/mg protein. All data were obtained from three independent biological replicates and are presented as mean ± standard deviation (*n* = 3).

### 2.5 Circular dichroism spectroscopy

Far-ultraviolet circular dichroism (CD) spectroscopy was performed to examine salt-dependent changes in the secondary structure and conformational stability of *Hh*MsrA and its mutants. Prior to CD measurements, purified proteins were buffer-exchanged into 20 mM phosphate buffer (PB, pH 8.0) using 10 kDa ultrafiltration centrifugal units. Samples were centrifuged at 4 °C and 4,000 × g for 20 min to remove the original filtrate. The retentate was resuspended in 15 mL of 20 mM PB (pH 8.0) and centrifuged under the same conditions for 30 min. This washing step was repeated once to ensure complete replacement of residual buffer components. The desalted proteins were collected and kept on ice prior to measurements, and the final filtrate was reserved as the blank control for baseline subtraction.

Protein samples were adjusted to a final concentration of 0.2 mg/mL in 20 mM PB (pH 8.0) and subjected to two salt conditions, including 0 M KCl (salt-free) and 3 M KCl (high-salt). CD spectra were recorded using a 1 mm path-length quartz cuvette with a sample volume of 300 μL on a CD spectropolarimeter (Bio-Logic, France). Spectra were collected over a wavelength range of 190–260 nm with a step size of 1 nm, an acquisition time of 1 s per step, and a slit width of 2 nm. Corresponding buffer spectra (final filtrates) under matched salt conditions were subtracted from protein spectra. Raw CD signals recorded in millidegrees (mdeg) were converted to mean residue ellipticity and are presented as ×10^3^ deg.·cm^2^·dmol^−1^ for subsequent analysis and plotting.

### 2.6 Molecular dynamics simulations

Three-dimensional structural models of *Hh*MsrA-WT and the indicated mutants were constructed using the SWISS-MODEL server (Waterhouse et al., 2018), with the 2.0 Å X-ray crystal structure of *Neisseria meningitidis* PilB MsrA in the reduced state (PDB ID: 3BQE) as the template (Ranaivoson et al., 2008). Detailed information on the model–template alignment, sequence coverage, and model-quality assessment is provided in Supplementary Figure S2. Molecular dynamics (MD) simulations were performed using GROMACS 2022.6 (Abraham et al., 2015) with the AMBER ff14SB protein force field implemented in the amber14sb_OL15 GROMACS force-field package (Maier et al., 2015). Each protein model was placed in a periodic cubic water box with a 10 Å buffer distance between the solute and the box boundary, and the system was solvated using TIP3P water molecules. Counterions were added as required to neutralize each system, after which the systems were prepared under nominal 0 M and 1 M KCl conditions. For the nominal 1 M KCl condition, the numbers of K^+^ and Cl^−^ ions were determined using the GROMACS genion tool based on the total volume of the periodic simulation box. The protein excluded volume was not subtracted from this calculation; therefore, 1 M refers to the nominal KCl concentration calculated from the total box volume rather than the effective concentration within the solvent-accessible volume. For the nominal 0 M condition, no additional KCl ion pairs were introduced beyond any counterions required for system neutralization. Energy minimization was performed using the steepest descent algorithm, followed by equilibration consisting of 500 ps NVT and 500 ps NPT. During NVT equilibration, the temperature was maintained at 300 K using the V-rescale thermostat with a coupling time constant of 0.1 ps. During NPT equilibration, the temperature was maintained at 300 K using the Nosé–Hoover thermostat with a coupling time constant of 1.0 ps, while the pressure was maintained isotropically at 1 bar using the Parrinello–Rahman barostat with a coupling time constant of 5.0 ps. Hydrogen-containing bonds were constrained using the LINCS algorithm. Long-range electrostatics were treated using the particle-mesh Ewald method, and the van der Waals interaction cutoff was set to 1.0 nm. Two independent 100-ns production simulations were performed for each system at 300 K and 1 bar using the V-rescale thermostat with a temperature-coupling time constant of 0.1 ps and the isotropic Parrinello–Rahman barostat with a pressure-coupling time constant of 2.0 ps. Trajectories were saved every 10 ps for analysis.

Trajectory analyses were performed using built-in GROMACS tools supplemented with Python scripts. All trajectory analyses were performed independently for the two production simulations. Root-mean-square deviation (RMSD) and root-mean-square fluctuation (RMSF) were calculated using built-in GROMACS tools (Abraham et al., 2015). For graphical presentation, the corresponding time- or residue-resolved profiles and summary values were averaged across the two independent simulations. For RMSD visualization, the replica-averaged profiles were subsequently smoothed using a Savitzky–Golay filter with a second-order polynomial and a 101-point window, corresponding to approximately 1 ns. The unsmoothed replica-averaged profiles were shown as lighter background traces. Salt bridges were identified using Visual Molecular Dynamics (VMD; Humphrey et al., 1996) by calculating, for each candidate residue pair and each frame of the 100-ns production trajectory, the minimum distance between acidic side-chain oxygen atoms (Asp/Glu) and basic side-chain nitrogen atoms (Arg/Lys). A salt bridge was considered to be formed when this minimum oxygen–nitrogen distance was ≤4.0 Å. Salt-bridge effective time was defined as the cumulative duration of all frames satisfying this criterion and was calculated by multiplying the total number of qualifying frames by the frame interval of 0.01 ns, irrespective of whether these frames were consecutive. Salt bridges with an effective time below 1% of the total simulation time were excluded from statistical summaries. Secondary-structure analysis was performed using Define Secondary Structure of Proteins (DSSP; Kabsch and Sander, 1983), and combined *α*-helix occupancy was quantified across residues 65–79 and 101–120, which correspond to the two principal helical segments harboring the Lys76 and Arg106 nodes, respectively. At each trajectory frame, combined helix occupancy was calculated as the total number of residues assigned as *α*-helix across the two segments divided by the total number of residues in both segments (35 residues).

## 3 Results and discussion

### 3.1 Global salt-bridge analysis reveals a salt-stabilized electrostatic network in *Hh*MsrA

**Figure 1:**
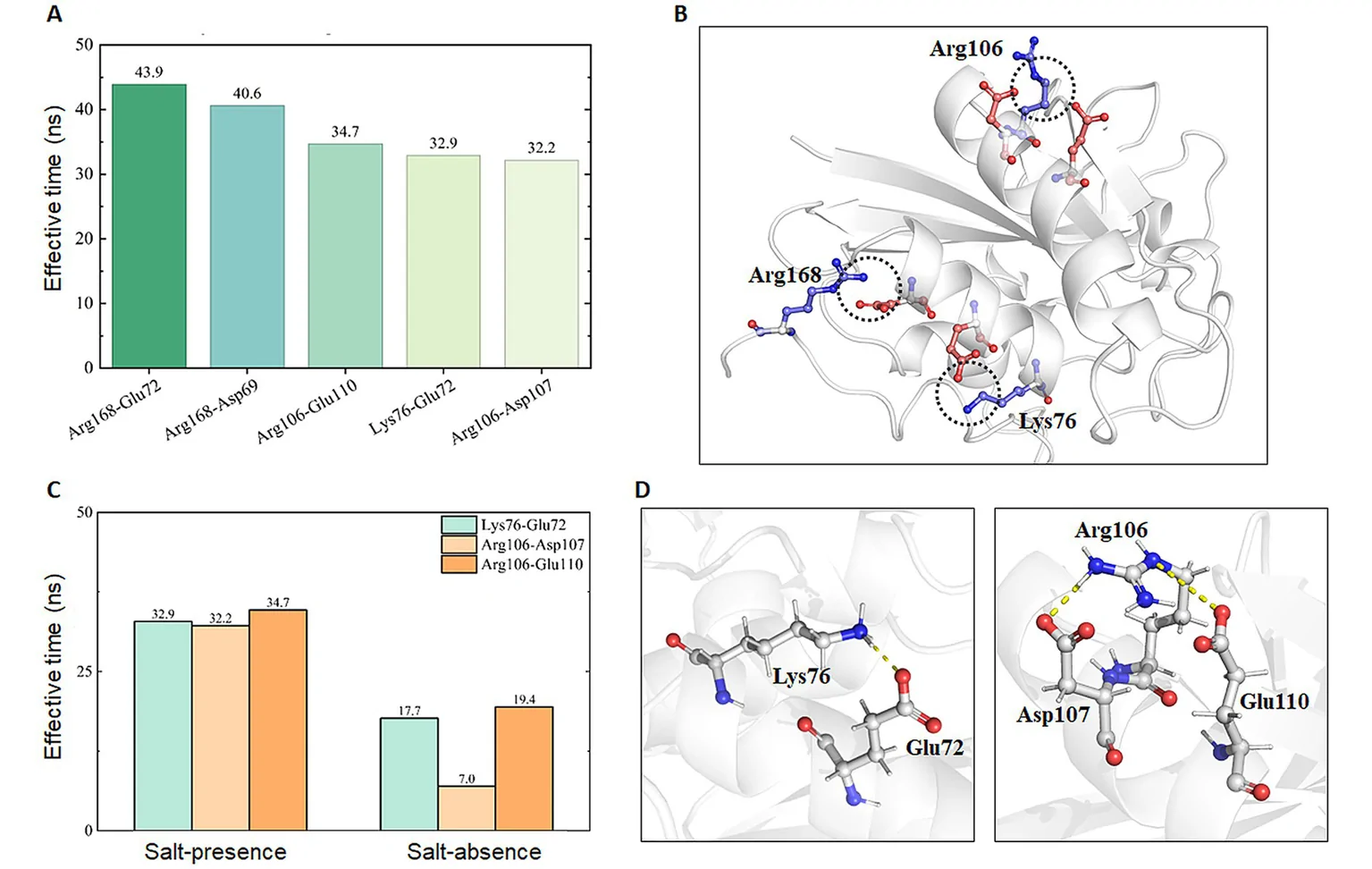
Global salt-bridge analysis and structural mapping in *Hh*MsrA. **(A)** Effective times (ns) of major salt bridges from MD simulations in the presence of KCl. **(B)** Mapping of key salt-bridge nodes (Lys76, Arg106, and Arg168) and their acidic partners onto the *Hh*MsrA structural model. **(C)** Effective-time comparison in the absence versus presence of KCl for Lys76-Glu72, Arg106-Glu110, and Arg106-Asp107. **(D)** Zoomed-in views of the Lys76- and Arg106-associated salt bridges in α-helical regions. Effective-time values in **(A)** and **(C)** represent means from two independent 100-ns MD simulations.

We first analyzed the global salt-bridge network under salt conditions using MD simulations. As shown in Figure 1A, three basic residues—Lys76, Arg106, and Arg168—each formed at least one long-lived salt bridge with a mean effective time exceeding 30 ns. Arg168, which was previously reported to contribute to *Hh*MsrA activity under high-salt conditions, formed persistent interactions with Asp69 and Glu72 (Zhou et al., 2024). Furthermore, Lys76 formed a stable interaction with Glu72 (32.9 ns), and Arg106 participated in two persistent salt bridges with Asp107 (32.2 ns) and Glu110 (34.7 ns). Structural mapping of these interactions onto the *Hh*MsrA model showed that Lys76, Arg106, and Arg168 are spatially distributed across the protein surface and together define major electrostatic nodes within the salt-bridge network (Figure 1B). These nodes converge on *α*-helical structural elements, with Lys76 located within the helix spanning residues 65–79 and Arg106 located within the helix spanning residues 101–120. Arg168 formed salt bridges with Asp69 and Glu72 near the 65–79 helix, potentially stabilizing the Lys76-containing helical region. Comparative analyses of halophilic proteins have shown that salt bridges are frequently organized into cooperative networks and can provide greater stabilization than isolated ion pairs (Nayek et al., 2014). At a different structural level, intersubunit salt-bridge clusters in *Haloarcula marismortui* malate dehydrogenase modulate salt-dependent oligomerization, whereas the major electrostatic nodes identified in *Hh*MsrA are intramolecular and converge on defined α-helical elements (Irimia et al., 2003). Because the functional role of Arg168 has been characterized in our previous study, the present work focuses on Lys76 and Arg106 to evaluate their contributions to salt-dependent activity.

To determine whether these Lys76- and Arg106-associated interactions were strengthened by salt, we compared their mean effective times in the absence and presence of KCl. The effective time of Lys76-Glu72 increased from 17.7 ns in the absence of KCl to 32.9 ns in the presence of 1 M KCl. Likewise, Arg106-Asp107 increased from 7.0 ns to 32.2 ns, and that of Arg106-Glu110 increased from 19.4 ns to 34.7 ns under the corresponding conditions (Figures 1C,D). Together with our previous identification of Arg168 as an important salt-bridge contributor (Zhou et al., 2024), these results suggest that multiple electrostatic nodes may contribute to the maintenance of the salt-adapted structural state of *Hh*MsrA. Accordingly, we next focused on Lys76 and Arg106 and performed systematic and combinatorial mutagenesis to assess their individual and combined contributions to salt-dependent activity.

### 3.2 Mutational analysis of Lys76 and Arg106 reveals charge-dependent effects on *Hh*MsrA activity

**Figure 2:**
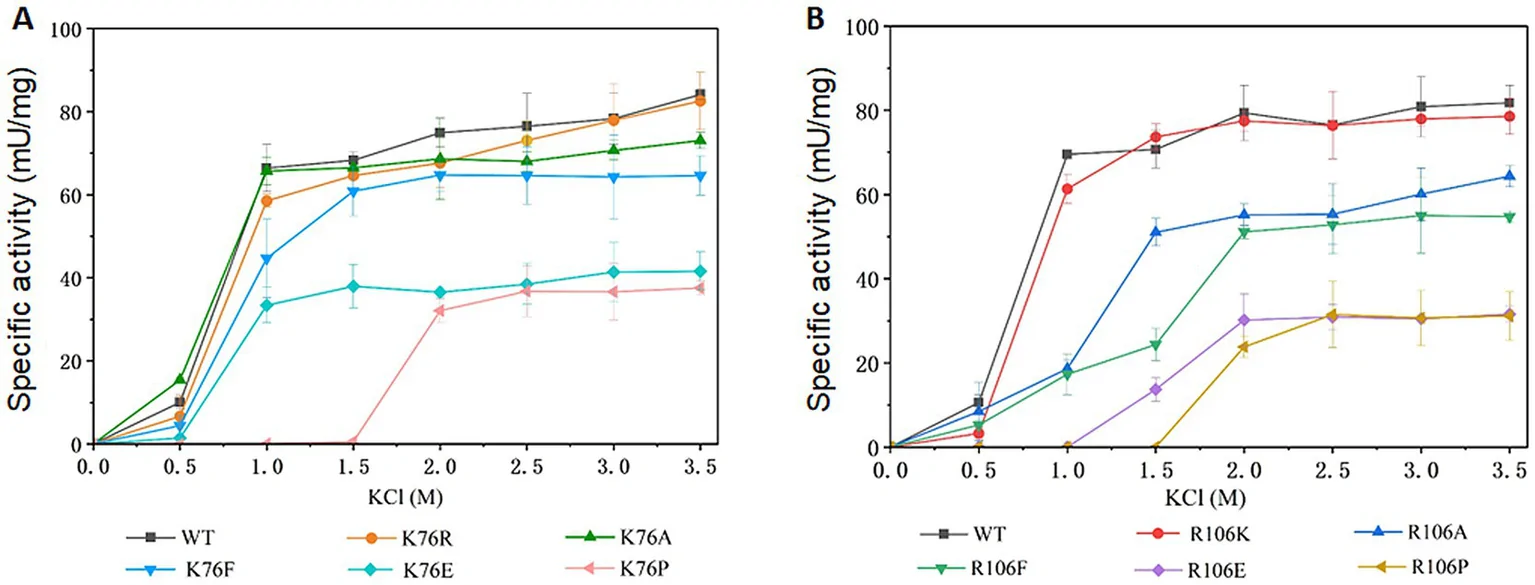
Mutational analysis of Lys76 and Arg106 in salt-dependent activity of *Hh*MsrA. **(A)** Specific activity of *Hh*MsrA-WT and Lys76 variants (K76R, K76A, K76F, K76E, and K76P) measured across a KCl gradient (0–3.5 M). **(B)** Specific activity of *Hh*MsrA-WT and Arg106 variants (R106K, R106A, R106F, R106E, and R106P) measured across a KCl gradient (0–3.5 M). Activities were determined using *L*-Met-O as substrate and are reported as specific activity (mU/mg). Data are shown as mean ± SD (*n* = 3).

To evaluate the functional contribution of Lys76, we generated a panel of site-directed mutants in which the basic Lys residue was replaced by a charge-conservative basic residue (Arg), a negatively charged residue (Glu), a hydrophobic residue (Ala), a conformationally restrictive residue (Pro), or an aromatic residue (Phe) (Figure 2A). All variants were successfully expressed upon IPTG induction and purified by Ni–NTA affinity chromatography (Supplementary Figure S1). The wild-type enzyme showed a typical salt-dependent activity profile, with no detectable activity at 0 M KCl, weak activity at 0.5 M KCl, and a marked increase from 1.0 M KCl onward, reaching a maximum at 3.5 M KCl (84.12 ± 1.20 mU/mg) (Figure 2A). The charge-conservative substitution K76R retained a wild-type-like response to increasing KCl concentrations and reached 82.58 ± 6.92 mU/mg at 3.5 M KCl, consistent with functional tolerance to a basic-to-basic replacement at this site. By comparison, K76A and K76F showed moderate reductions in maximal activity at 3.5 M KCl (73.06 ± 1.98 and 64.63 ± 4.69 mU/mg, respectively), whereas substitutions that do not preserve a basic residue caused larger decreases (K76E: 41.58 ± 4.59 mU/mg; K76P: 37.56 ± 1.69 mU/mg at 3.5 M KCl).

We next performed a parallel mutational analysis at Arg106 to test whether preserving a basic residue likewise supports *Hh*MsrA activity under high-salt conditions (Figure 2B). The charge-conservative mutant R106K largely preserved the wild-type behavior and exhibited a similar maximal activity at 3.5 M KCl (78.55 ± 4.29 mU/mg), indicating that maintaining a basic residue at this position is sufficient to sustain high-salt activity. By contrast, replacing Arg106 with substitutions that do not retain a basic residue reduced activity under high-salt conditions to varying extents. R106A and R106F reached lower maxima at 3.5 M KCl (64.34 ± 2.55 and 54.74 ± 1.37 mU/mg, respectively), whereas both R106E and R106P showed much lower activities at high salt, with maximal values of 31.47 ± 1.86 mU/mg (R106E) and 31.14 ± 5.75 mU/mg (R106P) at 3.5 M KCl. Overall, these results indicate that retaining a basic residue at positions 76 and 106 is important for maintaining robust activity at elevated KCl concentrations. Notably, Lys76 and Arg106 map to the 65–79 and 101–120 helices, respectively. The near-complete functional preservation after the Lys-Arg substitutions, contrasted with the stronger effects of charge reversal or proline substitution, indicates that positive charge is the principal requirement at these nodes, while side-chain geometry and compatibility with the local *α*-helical structure further modulate their stabilizing effects, consistent with the geometry-dependent contribution of intrahelical salt bridges reported previously (Meuzelaar et al., 2014; Meuzelaar et al., 2016). A comparable environment-dependent conformational effect has also been observed in the halophilic DNA ligase from *Haloferax volcanii*, in which K^+^ preferentially stabilizes a catalytically competent conformation (Ortega et al., 2011).

### 3.3 Combined mutations reveal cooperative support for salt-dependent *Hh*MsrA activity

**Figure 3:**
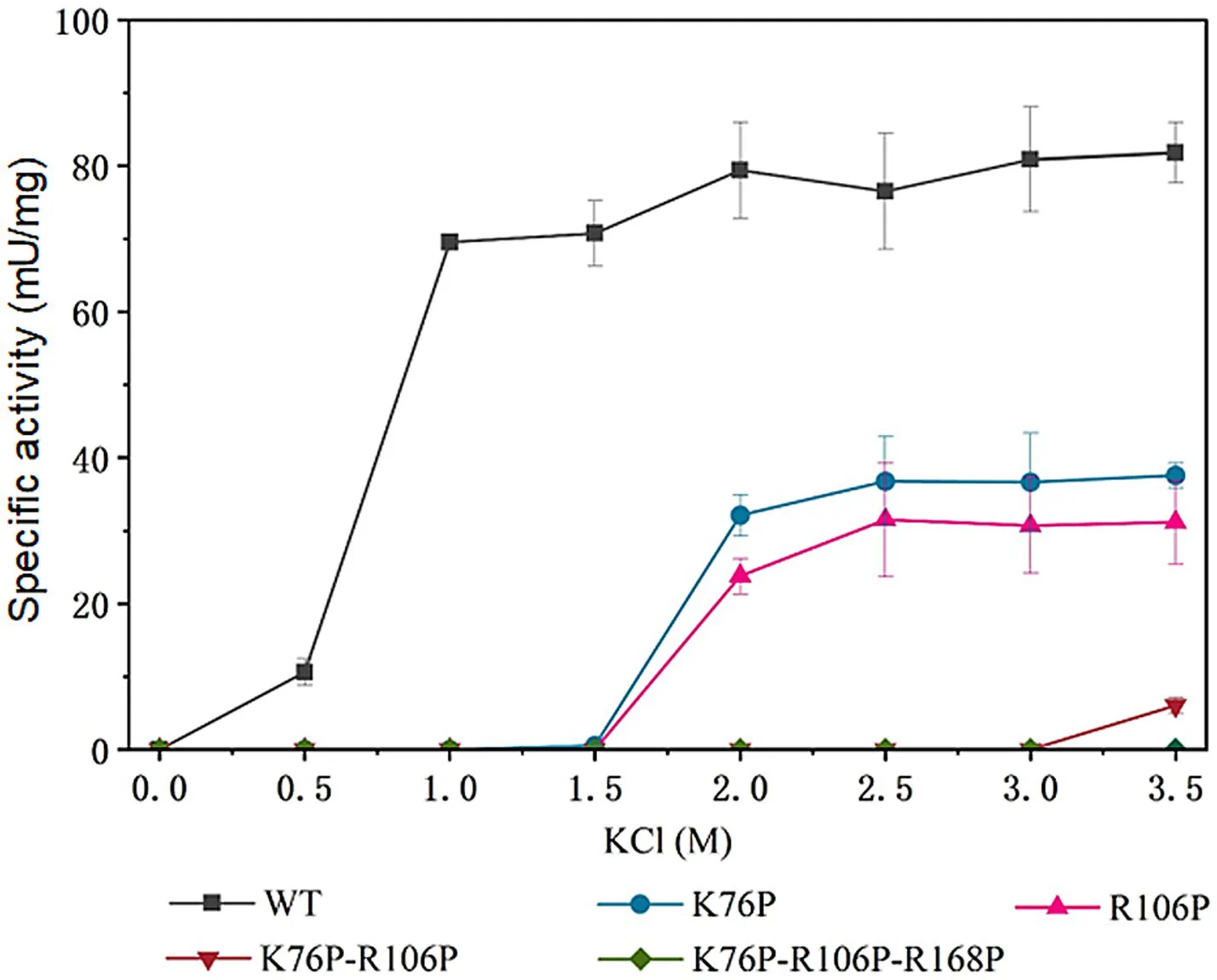
Combined proline substitutions markedly impair the salt-dependent activity of *Hh*MsrA. Specific activity of *Hh*MsrA-WT, single proline mutants (K76P and R106P), the double mutant (K76P–R106P), and the triple mutant (K76P–R106P–R168P) measured across a KCl gradient (0–3.5 M). Activities were determined using L-Met-O as the substrate and are reported as specific activity (mU/mg). Data are shown as mean ± SD (*n* = 3).

To assess the combined contributions of Lys76 and Arg106 to *Hh*MsrA activity under high-salt conditions, we constructed the double mutant (K76P–R106P). As shown in Figure 3, while the single mutants K76P and R106P retained measurable salt-dependent activity at high salinity, the double mutant exhibited a substantially greater reduction in activity across the KCl gradient. Specifically, K76P–R106P remained essentially inactive across 0–3.0 M KCl, and reached only 6.03 ± 1.09 mU/mg at 3.5 M KCl (approximately 10% of the wild-type activity) (Figure 3). The markedly greater loss of activity in K76P–R106P than in either single mutant supports cooperative, rather than independent, contributions of Lys76 and Arg106 to the electrostatic network maintaining the salt-adapted functional state. This interpretation is consistent with analyses of halophilic proteins showing that networked salt bridges can provide greater stabilization than isolated ion pairs (Nayek et al., 2014).

Building on the previously identified role of Arg168 in supporting *Hh*MsrA activity under high-salt conditions (Zhou et al., 2024), we next introduced R168P into the K76P–R106P background to generate the triple mutant (K76P–R106P–R168P). In contrast to the residual activity observed for the single mutants, the triple mutant displayed no detectable activity across the entire KCl range tested (Figure 3). The additional loss caused by R168P indicates that Arg168 provides residual electrostatic support when the Lys76- and Arg106-associated nodes are already disrupted. Thus, the double- and triple-mutant phenotypes support a threshold-like network behavior: partial disruption reduces but does not eliminate salt-dependent activity, whereas cumulative disruption of all three nodes abolishes the detectable response. Such background-dependent mutational effects are consistent with intramolecular coupling within protein interaction networks, in which the functional consequence of one substitution depends on the state of other interacting sites (Buda et al., 2023). Moreover, proline substitutions can directly perturb *α*-helical structure. Therefore, the pronounced activity loss of the double mutant and the complete loss of detectable activity in the triple mutant may partly reflect the intrinsic helix-destabilizing effect of proline, in addition to the cumulative disruption of salt-bridge-associated electrostatic interactions.

### 3.4 MD simulation reveals salt-bridge-dependent structural stability

**Figure 4:**
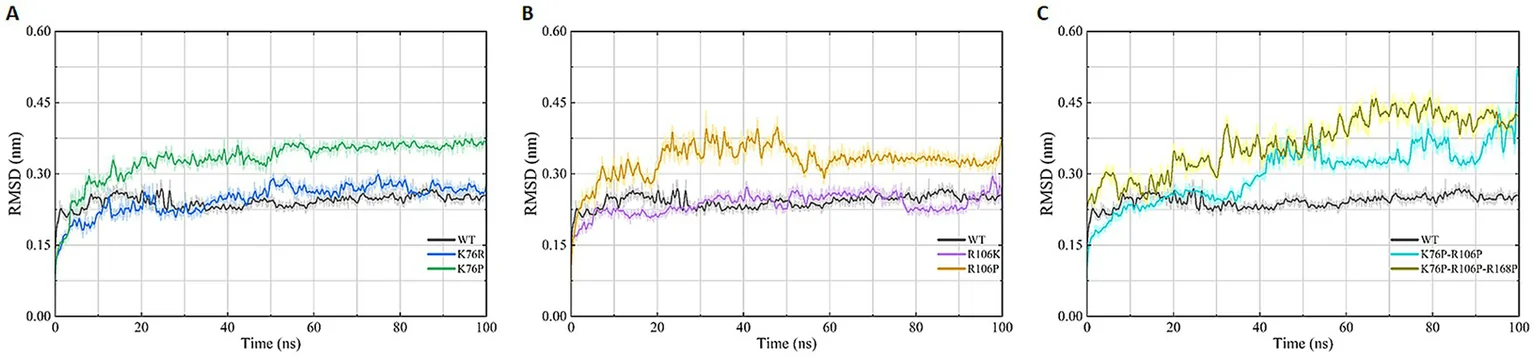
All-atom RMSD trajectories of *Hh*MsrA-WT and representative mutants at positions 76 **(A)** and 106 **(B)**, together with the combined mutants **(C)**, from MD simulations in the presence of KCl. The profiles represent mean values from two independent 100-ns MD simulations. The darker lines represent the smoothed replica-averaged RMSD profiles, whereas the lighter traces show the corresponding unsmoothed profiles.

To gain structural insight into the effects of charge-conservative versus proline substitutions, we performed MD simulations of *Hh*MsrA-WT, the single mutants K76R/K76P and R106K/R106P, as well as the combined mutants K76P–R106P and K76P–R106P–R168P under saline conditions. All-atom RMSD trajectories indicated that *Hh*MsrA-WT reached a stable plateau, fluctuating mainly around 0.22–0.28 nm over the course of the simulations (Figure 4), suggesting that the enzyme maintains a stable structural state under saline conditions. The charge-conservative mutants showed overall behavior similar to that of the WT, with K76R and R106K closely tracking the WT profile (Figures 4A,B), suggesting that basic-to-basic substitutions do not perturb the overall structural framework required for salt-dependent activity.

By contrast, both proline substitutions (K76P and R106P) exhibited elevated RMSD levels and larger fluctuations, remaining around ~0.4 nm and deviating clearly from the WT plateau (Figures 4A,B). Elevated conformational deviations were also observed when multiple salt-bridge-forming residues were simultaneously perturbed. The double mutant K76P–R106P remained above the WT baseline and showed increased late-stage fluctuations (Figure 4C). The triple mutant, which includes the previously characterized R168P substitution (K76P–R106P–R168P), also remained clearly above the WT baseline and displayed sustained fluctuations over much of the trajectory (Figure 4C). Although per-residue RMSF analysis showed that the terminal ~10 residues exhibited the largest fluctuations, elevated RMSF values were also evident in several internal regions, particularly in the combined mutants (Supplementary Figure S3). Consistently, RMSD analysis restricted to residues 1–160 retained the principal trends observed for the entire modeled region, indicating that the conformational deviations were not solely attributable to C-terminal mobility (Supplementary Figure S4). Taken together, these analyses indicate that proline substitution, particularly in combination, is associated with increased and regionally distributed conformational deviations of *Hh*MsrA under saline conditions, consistent with the graded reduction in activity observed for the corresponding mutants under saline conditions.

**Figure 5:**
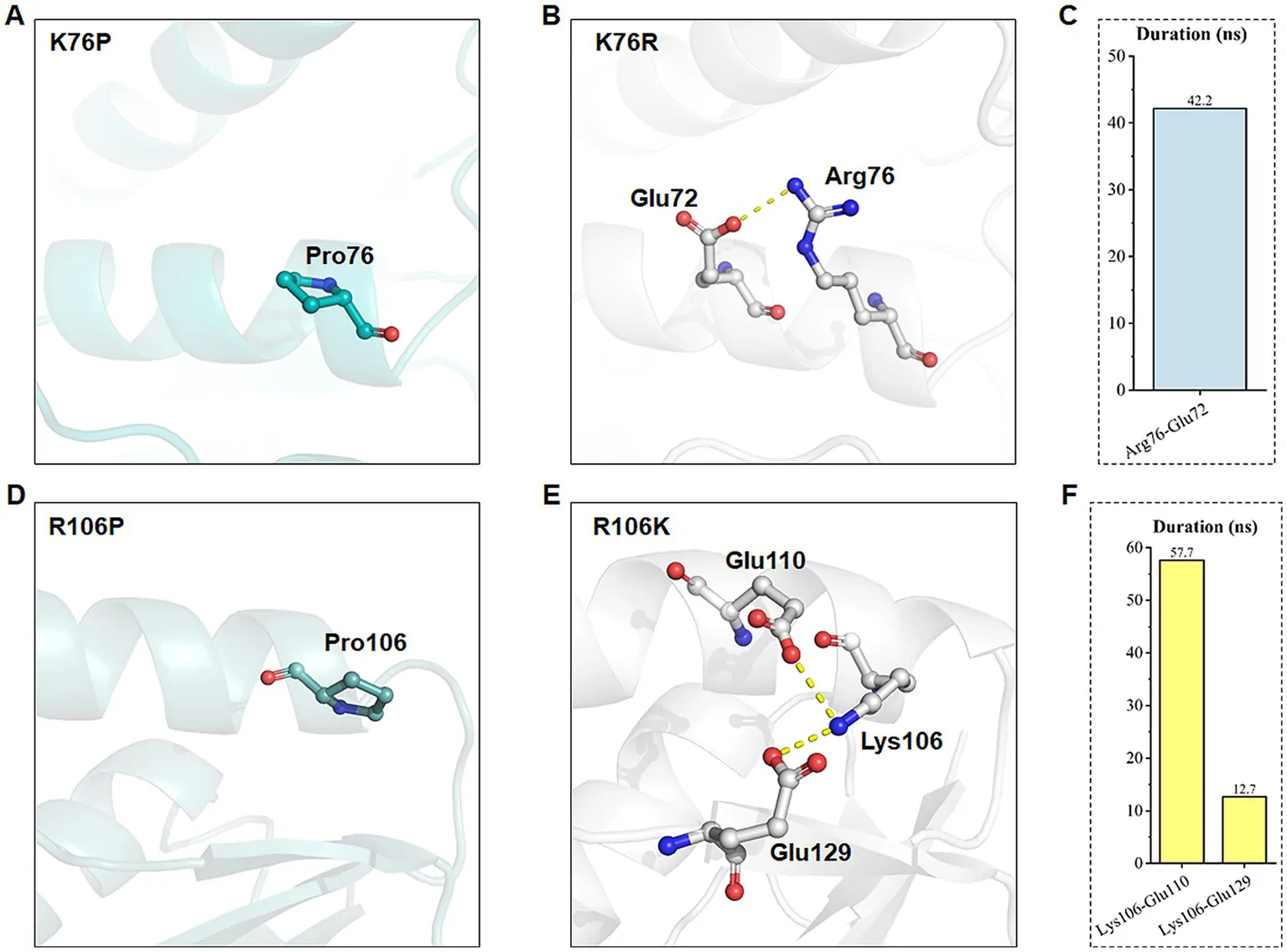
Site-resolved structural views and effective-time summaries of salt bridges at the Lys76 and Arg106 nodes from MD simulations under saline conditions. **(A,B)** Local views of the Lys76 site in variants of K76P **(A)** and K76R **(B)**. **(C)** Effective time of the Arg76-Glu72 ion pair in K76R. **(D,E)** Representative local views of the Arg106 site in variants of R106P **(D)** and R106K **(E)**. **(F)** Effective times of Lys106-centered ion pairs in R106K. Effective-time values in **(C)** and **(F)** represent means from two independent 100-ns MD simulations.

To connect the observed dynamic behavior with electrostatic interactions at the targeted sites, we quantified the persistence of the site-associated salt bridges centered on residues 76 and 106 (Figure 5). At the 76 site, the representative structural view showed that the proline substitution (K76P) abolished the local ion-pair geometry (Figure 5A), whereas preserving a basic side chain (K76R) maintains a stable Glu72-associated contact (Figure 5B). Consistently, K76R displayed an effective time of 42.2 ns for the Arg76-Glu72 ion pair (Figure 5C). At the 106 site, the representative structural view indicates that proline substitution (R106P) disrupts the salt-bridge-forming configuration at this site (Figure 5D), whereas the charge-conservative mutant (R106K) retains persistent electrostatic contacts (Figure 5E). In R106K, Lys106 engages Glu110 and also forms an additional contact with Glu129, with effective times of 57.7 ns and 12.7 ns, respectively (Figure 5F), and the combined contact time at residue 106 is comparable to that in *Hh*MsrA-WT (Figure 1A). Together, these site-resolved analyses indicate that maintaining a basic residue at the electrostatic nodes preserves persistent ion-pair interactions in the helical regions, whereas proline substitution disrupts these local electrostatic contacts, providing a possible structural explanation for the reduced high-salt activity of the corresponding mutants. More broadly, the association between persistent electrostatic contacts and conformational stability in *Hh*MsrA parallels findings in thermophilic proteins, where increased numbers and cooperative networks of salt bridges can rigidify protein structures at elevated temperatures (Kumar et al., 2000). The environmental stress differs, but in both cases electrostatic interactions help preserve a conformational state compatible with function.

### 3.5 Salt-induced helical organization revealed by circular dichroism and MD secondary-structure analysis

**Figure 6:**
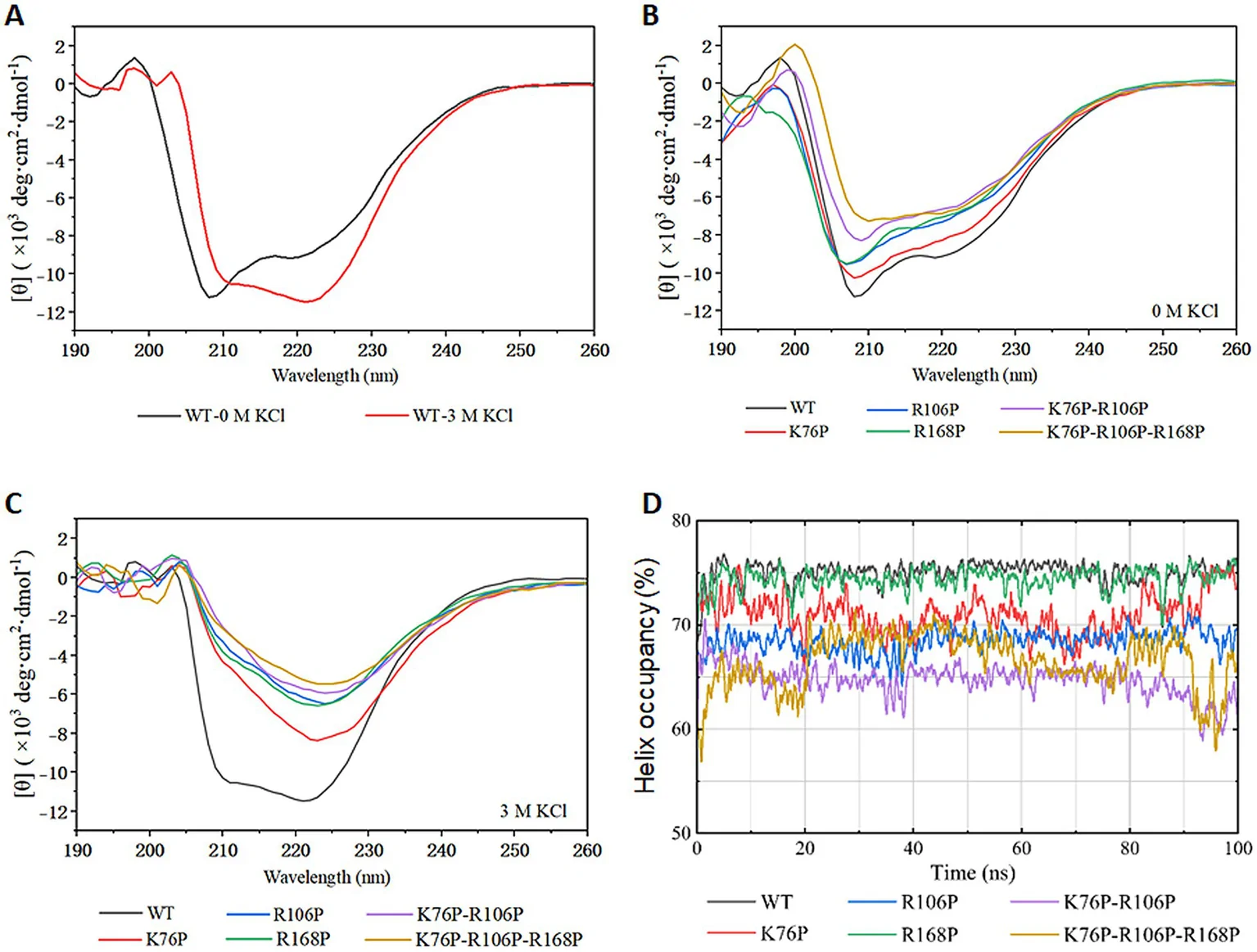
Circular dichroism (CD) spectra indicate a salt-dependent helical response of *Hh*MsrA and its dependence on salt-bridge-forming residues **(A)** Far-UV CD spectra of *Hh*MsrA recorded in the absence and presence of KCl, showing a pronounced salt-induced change in the helical region, with a marked increase near 222 nm **(B)** CD spectra of *Hh*MsrA and the mutant series recorded under salt-free conditions **(C)** CD spectra of *Hh*MsrA and the mutant series recorded under high-salt conditions, showing progressively weakened helical signatures in the mutants relative to *Hh*MsrA **(D)** Combined DSSP-based helix occupancy of the salt-bridge-associated helices spanning residues 65–79 and 101–120 during MD trajectories under saline conditions. The profiles represent mean values from two independent 100-ns MD simulations.

Far-UV CD spectra were first recorded for *Hh*MsrA in the absence and presence of salt to define the salt-dependent structural response. As shown in Figure 6A, *Hh*MsrA exhibited a pronounced salinity-dependent change in the *α*-helical region. Notably, the helical signal near ~222 nm increased markedly in the presence of KCl, and the band around ~208 nm showed a subtle shift and reshaping. These spectral changes indicate that high salinity is associated with enhanced helical organization in *Hh*MsrA. Consistent with our previous observation that *Hh*MsrA shows no detectable activity in the absence of KCl but exhibits markedly increased activity in its presence, the pronounced structural response observed here supports the existence of a distinct high-salt structural state compatible with catalytic activity (Ozturk et al., 2020; Zhou et al., 2024).

We next compared the CD spectra of *Hh*MsrA-WT and the mutant series in the absence of KCl to assess the baseline structural effects of perturbing salt-bridge-forming residues. As shown in Figure 6B, the overall spectral profiles of the mutants remained broadly similar to that of *Hh*MsrA-WT, indicating that these variants retain a comparable folded framework under salt-free conditions. However, the magnitude of the helical-region signals was consistently reduced across the mutant series, and the attenuation became progressively more pronounced as additional salt-bridge-forming residues were perturbed, with the combined mutants showing the weakest helical signatures. These results indicate that the key basic residues Lys76, Arg106, and Arg168 contribute to maintaining helical structural features even in the absence of KCl, thereby providing a pre-organized scaffold for the subsequent structural response to salinity.

In the presence of KCl, the differences between *Hh*MsrA-WT and the mutant series became much more evident. As shown in Figure 6C, *Hh*MsrA displayed the strongest helical response, whereas all mutants showed markedly reduced helical signals in the same spectral region. The attenuation intensified with increasing mutational burden, and the combined mutants exhibited the weakest helical signatures under salt conditions. Together, these results indicate that the key basic residues Lys76, Arg106, and Arg168 contribute to the salt-dependent helical organization of *Hh*MsrA. The salt-bridge interactions formed by these residues appear to provide essential electrostatic support for this high-salt structural state. Disrupting these electrostatic nodes progressively compromises the salt-dependent helical strengthening, consistent with their importance in maintaining the structural state required for activity under high-salt conditions. Notably, Lys76 and Arg106 reside on *α*-helical segments spanning residues 65–79 and 101–120, respectively, and these two helices contain the major salt-bridge nodes identified by MD. This helix localization provides a direct structural context for why perturbing salt-bridge-forming residues preferentially weakens the helical response detected by CD (Meuzelaar et al., 2016; Batchelor et al., 2019).

To further assess the impact of salt-bridge-forming residues on local helical organization, we analyzed the MD trajectories using DSSP and quantified the overall helix occupancy of the two salt-bridge-associated regions spanning residues 65–79 and 101–120 under saline conditions (Figure 6D). *Hh*MsrA-WT maintained relatively stable helix occupancy over time. In contrast, proline substitutions at Lys76 and Arg106 reduced overall helix persistence and increased temporal fluctuations. The double mutant exhibited the lowest overall helix occupancy, whereas the triple mutant showed more pronounced temporal fluctuations despite maintaining a higher overall occupancy than the double mutant. Together, these DSSP results further support the importance of the salt-bridge network in stabilizing the overall helical organization of the two analyzed regions spanning residues 65–79 and 101–120 in *Hh*MsrA under saline conditions, particularly within the salt-bridge-associated helices spanning residues 65–79 and 101–120, in agreement with the helical response observed by CD (Lee et al., 2014; Nayek et al., 2014). More broadly, a rigidifying salt bridge in thermophilic acylphosphatase restricts active-site flexibility and favors catalytic activity at elevated temperatures, whereas the electrostatic network in *Hh*MsrA appears to stabilize salt-dependent helical organization under saline conditions (Lam et al., 2011).

## 4 Conclusion

In this study, we show that a small set of salt-bridge-forming residues contributes to the maintenance of a salt-adapted structural state required for *Hh*MsrA activity. Systematic mutagenesis demonstrated that retaining a basic residue at positions 76 and 106 is important for robust salt-dependent activity, whereas substitutions that remove this basic character reduce activity under high-salt conditions. The most severe losses occurred when multiple sites were perturbed simultaneously. Structural mapping further indicated that these salt-bridge residues converge on two *α*-helical segments, residues 65–79 and 101–120, linking site-specific electrostatic contacts to the stability of a helix-based framework. Circular dichroism revealed a strong salt-dependent helical response that was progressively weakened as salt-bridge-forming residues were perturbed, and MD-based analyses supported reduced helical stability within the salt-bridge-associated segments under saline conditions. Together, these findings provide a mechanistic basis for how enzymes from halophilic microorganisms couple salinity to catalytic competence through salt-bridge-mediated stabilization of a salt-adapted structural state. Building on the previously characterized Arg168-associated salt bridges, the present study experimentally evaluated two additional long-lived salt-bridge nodes centered on Lys76 and Arg106. Nevertheless, additional residues may contribute to the salt-dependent structural stability of *Hh*MsrA through transient ion-pair interactions or other electrostatic mechanisms. Future analyses integrating surface acidic-residue distribution, electrostatic potential, sequence conservation, and extended MD simulations may identify additional determinants of halophilic adaptation.

## Data Availability

The raw data generated in this study are available from the corresponding author upon reasonable request.
