# Supplementary material for: Investigation of salt-bridge interactions underlying salt activation of halophilic methionine sulfoxide reductase A
Source: Front Microbiol. 2026 Aug 24;17:1907662. doi: 10.3389/fmicb.2026.1907662 (PMC13561113; doi:10.3389/fmicb.2026.1907662)
Supplement: Supplementary file 1 [file Data_Sheet_1.PDF]

Supplementary Material

1 Supplementary Figures and Tables

1.1 Supplementary Figures

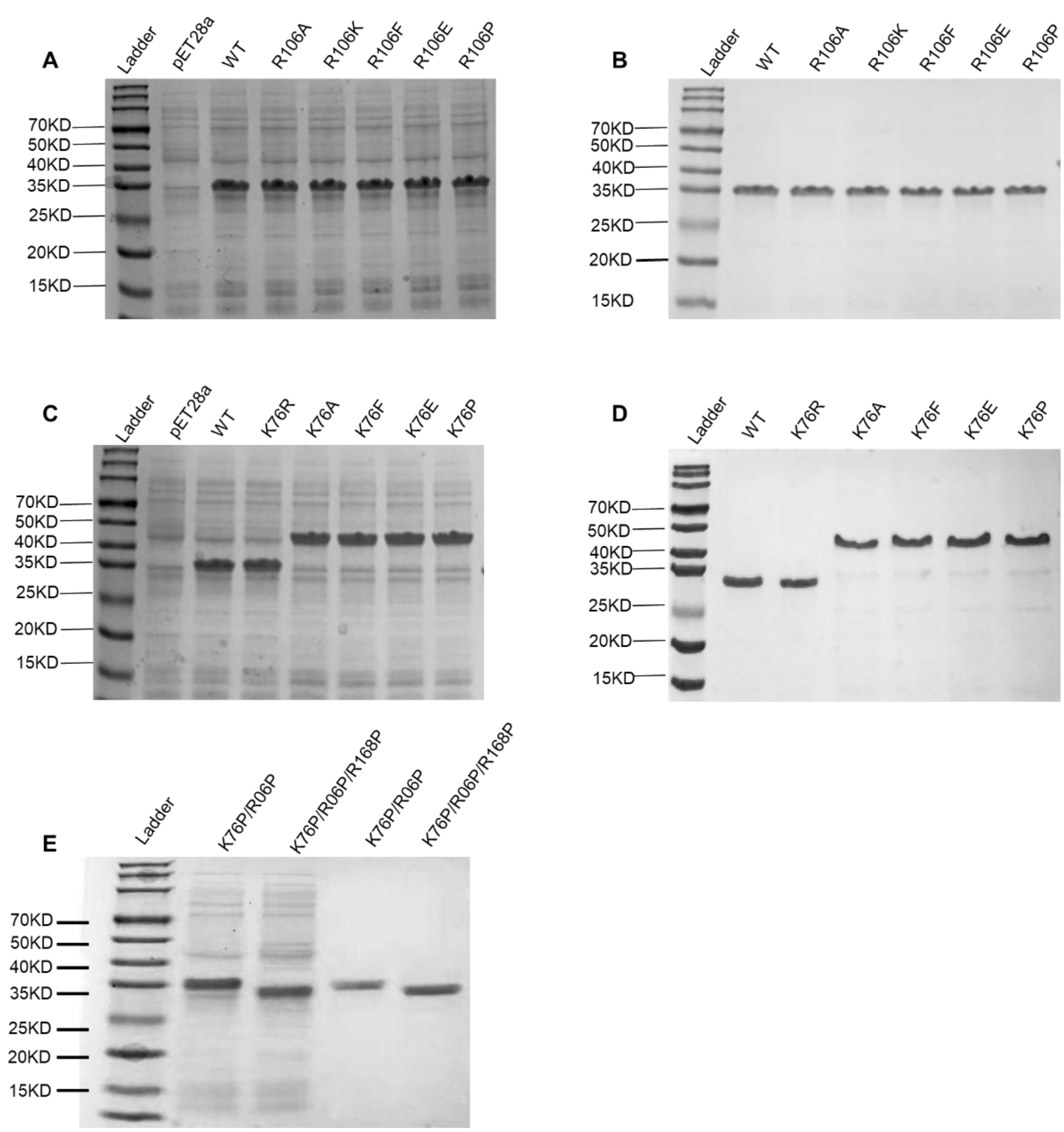

Supplementary Figure 1. SDS-PAGE gel analysis of the recombinant expression of *Hh*MsrA and its variants. 20 µg of soluble total proteins and 3 µg of pure enzymes were subjected to SDS-PAGE gel electrophoresis.

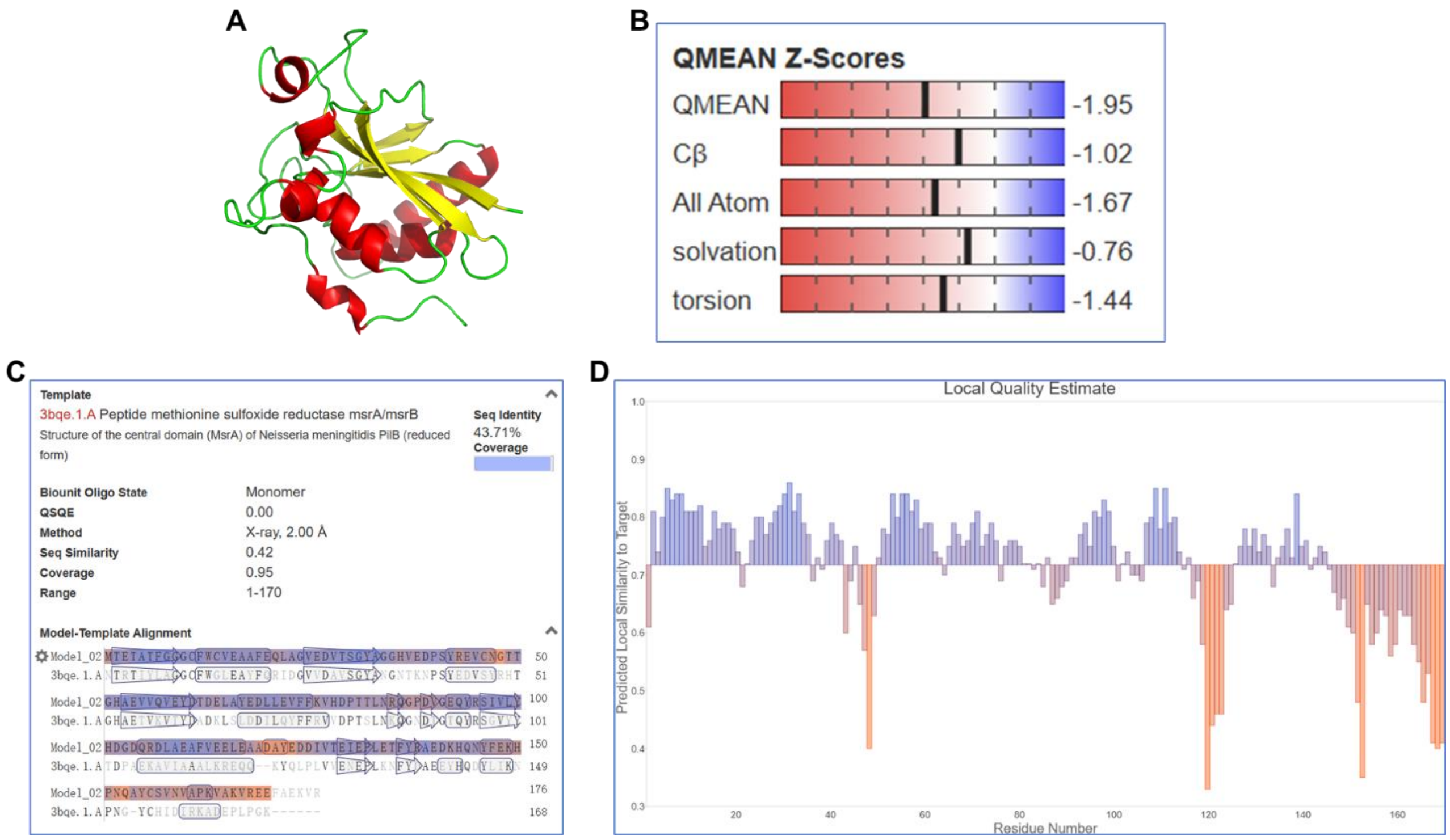

Supplementary Figure S2. Homology modeling and quality assessment of *Hh*MsrA. (A) Overall structural model of *Hh*MsrA generated using the SWISS-MODEL server. (B) Summary of the template and model-quality parameters. (C) Model–template sequence alignment between *Hh*MsrA and the reduced MsrA structure from *Neisseria meningitidis* PilB (PDB ID: 3BQE). (D) Per-residue local quality estimates for the predicted model.

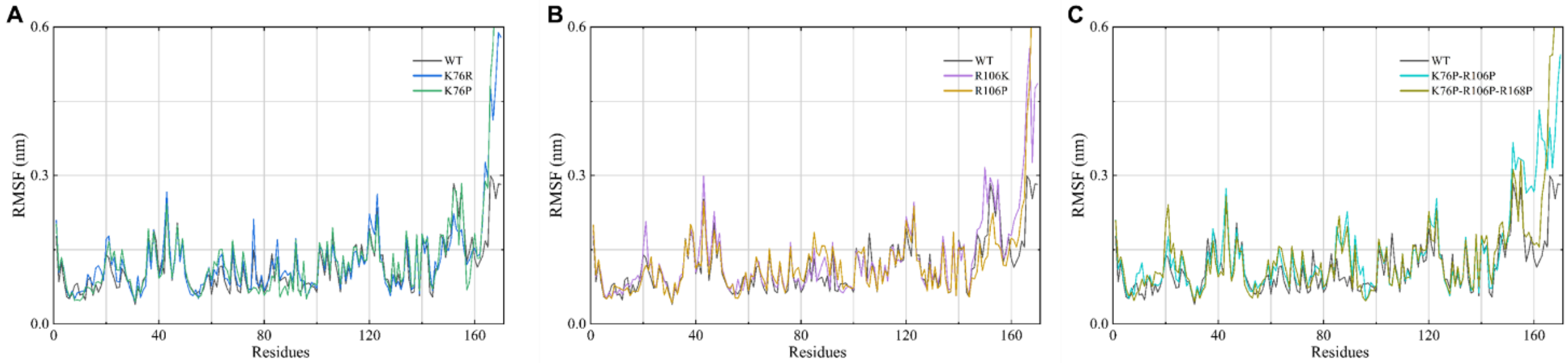

Supplementary Figure S3. Per-residue RMSF profiles of *Hh*MsrA-WT and the indicated mutants under saline conditions. (A) *Hh*MsrA-WT, K76R, and K76P. (B) *Hh*MsrA-WT, R106K, and R106P. (C) *Hh*MsrA-WT, K76P–R106P, and K76P–R106P–R168P. The profiles represent the mean values obtained from two independent 100-ns MD simulations.

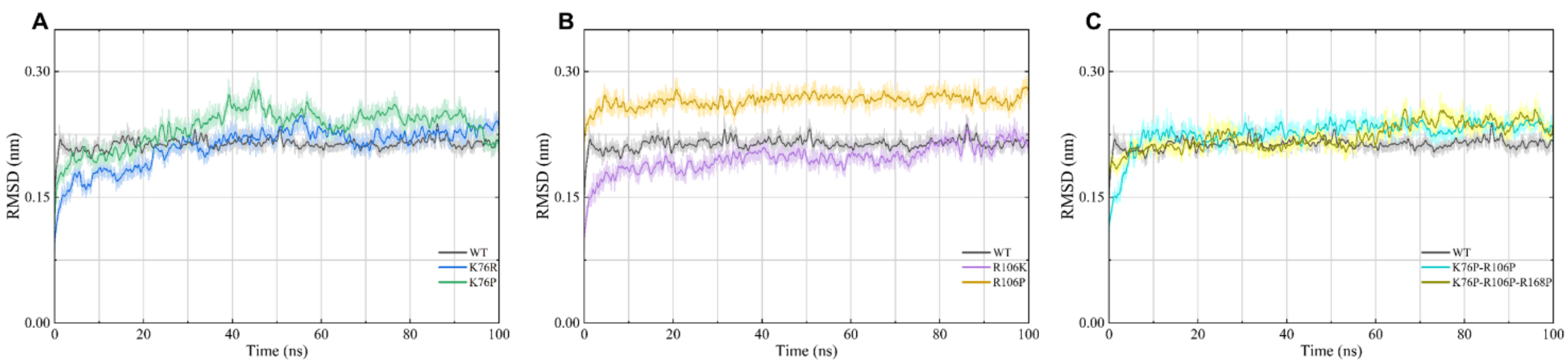

Supplementary Figure S4. RMSD profiles of residues 1–160 in *Hh*MsrA-WT and the indicated mutants under saline conditions. (A) *Hh*MsrA-WT, K76R, and K76P. (B) *Hh*MsrA-WT, R106K, and R106P. (C) *Hh*MsrA-WT, K76P–R106P, and K76P–R106P–R168P. The profiles represent the mean values obtained from two independent 100-ns MD simulations. The darker lines represent the smoothed replica-averaged RMSD profiles, whereas the lighter traces show the corresponding unsmoothed profiles.

1.2 Supplementary Tables

Supplementary Table S1 Oligonucleotide primers used in this study

Primer Name	Sequence (5′→3′)
R106K	F: CGACGGCGACCAGAAAGACCTCGCGGAGGC
	R: GCCTCCGCGAGGTCTTTCTGGTCGCCGTCG
R106F	F: CGACGGCGACCAGTTTGACCTCGCGGAGGC
	R: GCCTCCGCGAGGTCAAACTGGTCGCCGTCG
R106A	F: CGACGGCGACCAGGCGGACCTCGCGGAGGC
	R: GCCTCCGCGAGGTCCGCCTGGTCGCCGTCG
R106E	F: CTACCACGACGGCGACCAGGAAGACCTCGCGGAGGCGTTC
	R: GAACGCCTCCGCGAGGTCTTCCTGGTCGCCGTCGTGGTAG
R106P	F: CGACGGCGACCAGCCGGACCTCGCGGAGGC
	R: GCCTCCGCGAGGTCCGGCTGGTCGCCGTCG
K76R	F: CGAAGTGTTCTTCCGTGTCCACGACCCGAC
	R: GTCGGGTCGTGGACACGGAAGAACACTTCG
K76F	F: CGAAGTGTTCTTCTTTGTCCACGACCCGAC
	R: GTCGGGTCGTGGACAAAGAAGAACACTTCG
K76A	F: CTCGAAGTGTTCTTCGCGGTCCACGACCCGAC
	R: GTCGGGTCGTGGACCGCGAAGAACACTTCGAG
K76E	F: CGAAGTGTTCTTCGAAGTCCACGACCCGAC
	R: GTCGGGTCGTGGACTTCGAAGAACACTTCG
K76P	F: CTCGAAGTGTTCTTCCCGGTCCACGACCCGAC
	R: GTCGGGTCGTGGACCGGGAAGAACACTTCGAG

Supplementary Table S2 Replica-resolved effective times of the key salt bridges shown in Figures 1 and 5 obtained from two independent 100-ns MD simulations

Sample	KCl condition	Salt bridge	Replica 1 (ns)	Replica 2 (ns)	Mean (ns)
*Hh*MsrA-WT	1 M	Arg168-Glu72	30.0	57.8	43.9
		Arg168-Asp69	46.4	34.8	40.6
		Arg106-Glu110	30.3	39.1	34.7
		Lys76-Glu72	35.6	30.2	32.9
		Arg106-Asp107	30	34.4	32.2
	0 M	Arg106-Glu110	24.5	14.3	19.4
		Lys76-Glu72	26.6	8.8	17.7
		Arg106-Asp107	8.8	5.2	7
*Hh*MsrA-R106K	1 M	Lys106-Glu110	42.6	72.7	57.7
		Lys106-Glu129	25.4	0.0	12.7
*Hh*MsrA-K76R	1 M	Arg76-Glu72	34.1	50.2	42.2
